# Survey of Spanish general practitioners’ attitudes toward management of sore throat: an internet-based questionnaire study

**DOI:** 10.1186/s12875-017-0597-1

**Published:** 2017-02-14

**Authors:** Carl Llor, Isabel Vilaseca, Eduardo Lehrer-Coriat, Xavier Boleda, José L. Cañada, Ana Moragas, Josep M. Cots

**Affiliations:** 1 Primary Healthcare Centre Via Roma, Barcelona, Spain; 2 Department of Otorhinolaringology, Hospital Clínic of Barcelona, Barcelona, Spain; 3 Faculty of Medicine, University of Barcelona, Barcelona, Spain; 4 Pharmacy Arizcun (Group on respiratory diseases, Sociedad Española de Farmacia Comunitaria), Sant Pere de Ribes, Spain; 5 Primary Healthcare Centre Algorta (Group on Infectious Diseases SEMERGEN), Getxo, Vizcaya Spain; 6 Primary Healthcare Centre Jaume I, University Rovira i Virgili, Tarragona, Spain; 7 Primary Healthcare Centre La Marina (Group on Infectious Diseases, semFYC), University of Barcelona, Barcelona, Spain

**Keywords:** Antibiotics, Sore throat, Rational use of antibiotics, Spain, Primary care

## Abstract

**Background:**

The management of sore throat varies widely in Europe. The objective of this study was to gain insight into clinicians’ perceptions on the current management of sore throat in Spain.

**Methods:**

Cross-sectional, internet-based questionnaire study answered from July to September 2013. General practitioners (GPs) affiliated with the two largest scientific societies of primary care were invited to participate in the study. Questions were asked about physician knowledge, the use of current national guidelines for sore throat management, and management in two clinical scenarios, depicting a young adult with sore throat and: 1. cough, coriza with or without fever, and 2. fever without cough and coriza.

**Results:**

The questionnaire was completed by 1476 GPs (5%) and 12.7% declared using rapid antigen detection tests. Antibiotics were considered by 18.8% of the GPs in the first scenario and by 32% in the second scenario (*p* < 0.001). The antibiotics most commonly mentioned by GPs were amoxicillin and amoxicillin + clavulanate (52.7 and 31.2%, respectively) whereas penicillin V was only prescribed in 11.9% of the cases. The drugs most commonly considered in both scenarios were analgesics and anti-inflammatory drugs. Antitussives, decongestants and expectorants were more commonly prescribed in cases of suspected viral infection (*p* < 0.001).

**Conclusions:**

GPs have misconceptions as to the indications for using rapid antigen detection tests and prescribing drugs in the management of sore throat. These results suggest that guidelines are seldom followed since one in five GPs declared giving antibiotics for patients with a suspected viral infection and the use of second-choice antibiotics seems considerable.

## Background

**Fig. 1:**
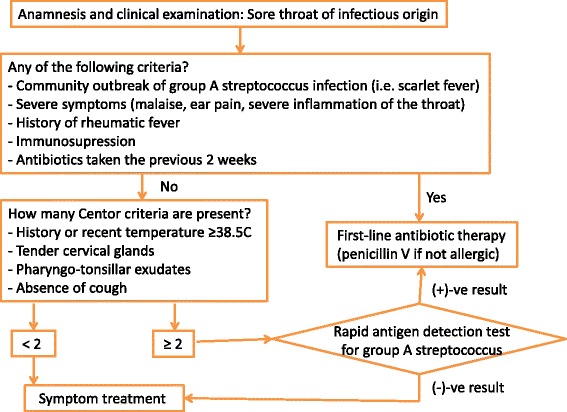
Recommended management of acute pharyngitis in Spain (based on reference 4)

Sore throat is one of the most frequent causes of visits to primary care and accounts for approximately 15% of general practitioner (GP) visits due to an infectious disease [1]. Group A β-haemolytic streptococcus (GAS) causes about 10% of the episodes of pharyngitis in adults [2]. Patients with sore throat due to GAS commonly present sore throat, tender cervical adenopathy, raised temperature, tonsillar exudate, and absence of cough; criteria that were identified by Centor et al. more than three decades ago [3]. However, none of these findings is specific for streptococcal pharyngitis. Microbiological culture of a throat swab remains the gold standard for the diagnosis of acute GAS pharyngitis. However, obtaining culture results takes at least 2 days, thereby limiting its utility in primary care. This drawback in obtaining results from cultures makes rapid antigen detection tests from throat swabs as recommended method in national guidelines for the rapid identification of GAS infection (Fig. 1) [4].

Appropriate diagnosis and treatment of GAS pharyngitis is crucial for preventing complications, such as peritonsillar abscess and acute rheumatic fever, and reducing the acute morbidity associated with the illness [5]. Conversely, improper diagnosis is associated with unnecessary antibiotic prescriptions and increased health care costs and contributes to the development of bacterial resistance [6]. Although only 10% of the cases require antibiotic treatment, approximately 60% of adults are currently treated with these drugs in Western countries [7, 8]. In a recent paper carried out in Spain, we observed that if GPs had appropriately used rapid antigen detection tests and had strictly followed current guidelines, 84.5% of the antibiotics prescribed for pharyngitis would have been saved [9]. Apart from diagnostic uncertainty other important factors influencing over-prescription of antibiotics for sore throat are patients’ expectations of antibiotics, and physicians’ assumptions regarding these expectations [10–12]. Local guidelines recommend the prescription of penicillin V for suspected GAS pharyngitis in patients otherwise not allergic to β-lactams [4], since it is a well-tolerated, inexpensive, narrow-spectrum antibiotic for which isolates of penicillin-resistant GAS have not yet been described, despite having been used for more 70 years. This is apparently because these microorganisms are intolerant to mutations that reduce the affinity of penicillin-binding proteins [13].

Postal surveys of GPs have traditionally been used to evaluate their knowledge, views and attitudes about medical conditions. The potential to use the internet as a research tool is growing as more GPs use Internet more actively [14]; and now nearly all the GPs in Spain have access to internet, at least in their workplace. This study was aimed to know GPs’ knowledge, beliefs, and how they manage sore throat by means of an internet-based questionnaire that was available online in the two largest scientific societies of primary care in this country.

## Methods

A cross-sectional survey among a sample of the Spanish GPs was conducted from July to September 2013, using an internet-based survey. The questionnaire was designed by a Spanish panel of GPs, ear-nose-and-throat specialists and pharmacists. A total of eight questions about physician knowledge and use of current national guidelines for management of sore throat with sub-items within the domains of the questions were included in the final instrument. GPs were also asked to state how they would manage sore throat in a young person with sore throat in two clinical scenarios: scenario A also depicted cough, coriza, and with or without fever; and scenario 2 also presented fever, but without cough and coriza. The internet-based questionnaire software required responders to answer a question with sub-items before being able to continue to the next question. Participants were able to respond the questionnaire only once. The inquiry was available online in two scientific society websites: *Sociedad Española de Medicina de Familia y Comunitaria* (SemFYC; www.semfyc.es) and *Sociedad Española de Médicos de Atención Primaria* (Semergen; www.semergen.es). Both societies account for more than 90% of the GPs affiliated in Spain, with a total of approximately 29,500 members. All responder answers were automatically entered into a data file which was checked for accuracy by two independent researchers. Descriptive and bivariate analyses were carried out. Data were analysed using SPSS (version 19.0).

## Results

A total of 1476 GPs completed the questionnaire (5%). The answers of the first questions are detailed in Table 1. Rapid antigen detection tests were declared to be used in 12.7% of the cases of sore throat. GPs stated that the incidence of streptococcal infection is observed in 20.6% of the cases. In suspected streptococcal pharyngitis, 175 GPs considered penicillin as the first-choice antibiotic (11.9%). The antibiotic most commonly mentioned as being prescribed by Spanish GPs for patients with sore throat was amoxicillin (778 cases, 52.7%), followed by amoxicillin + clavulanate (461 cases, 31.2%). Table 2 describes the treatment used by GPs in the two clinical scenarios. Thirty-two percent of the respondents endorsed the view that antibiotics are effective in treating pharyngeal infections in the second scenario; however, 18.8% of the respondents believed that antibiotics are also effective in treating suspected viral infections, as depicted in scenario A.

**Table 1:** Questionnaire answered by GPs regarding management of adult patients with sore throat (*n* = 1476)

Question	Mean (SD)
What percentage of patients with sore throat do you visit in overall in your consultation in one year?	27.9 (21.8)
Regarding patients with sore throat, what percentage do you suppose is caused by a bacterial infection?	20.6 (14.4)
In what percentage of patients suspected of having streptococcal infection do you perform a rapid antigen detection test?	12.7 (27.3)
What antibiotic do you prescribe in suspected streptococcal pharyngotonsillitis?	
- Penicillin V	175 (11.9)
- Amoxicillin	778 (52.7)
- Amoxicillin and clavulanate	461 (31.2)
- Azithromycin	52 (3.5)
- Other antibiotics	10 (0.7)

*SD* standard deviation

**Table 2:** Treatment that would be prescribed in two clinical scenarios of sore throat stated by the 1476 respondents

Drug prescribed	Scenario A	Scenario B	*P*
No treatment at all	3.0 (10.1)	2.4 (9.3)	NS
Analgesics	56.8 (34.4)	57.0 (35.5)	NS
Non-steroidal anti-inflammatory drugs	53.5 (31.6)	55.6 (31.8)	NS
Antibiotics	18.8 (22.8)	32.0 (32.6)	<0.001
Antitussives	20.3 (26.2)	2.0 (8.8)	<0.001
Decongestants	11.1 (19.4)	1.6 (7.0)	<0.001
Expectorants and/or mucolytics	19.9 (26.1)	2.4 (8.9)	<0.001
Phytotherapy	3.6 (14.4)	3.0 (13.2)	NS
Antiseptics	3.2 (12.5)	3.5 (12.9)	NS

Scenario A: young adult with the presence of sore throat, cough, coriza, and with or without fever; scenario B: young adult with the presence of sore throat, fever, and without cough and coriza

More than 50% of GPs usually prescribe analgesics and/or non-steroidal anti-inflammatory drugs, with ibuprofen being the most commonly prescribed (96.1%), followed by naproxen (1.8%) and diclofenac (1%). Daily doses of 1800 mg or higher of ibuprofen were recommended by 1200 out of the 1419 of the GPs who used ibuprofen (84.6%). Other treatments, such as expectorants, mucolytics and decongestants were more commonly used for patients with suspected viral infection (*p* < 0.001), as is shown in Table 2.

## Discussion

These data reveal important misconceptions that GPs, who are members of the two largest scientific societies of general practice in Spain, have about the appropriate indications for using antibiotics in adult patients with sore throat. Nearly 19% of GPs would prescribe antibiotics in the first scenario, depicting a patient with a suspected viral sore throat, with 0 or 1 Centor score, while 32% would do the same for a patient with an uncertain-origin infection, presenting 2 Centor criteria. It is remarkable that despite the fact that group A streptococcus remains universally susceptible to penicillin this antibiotic was only preferred by 12% of the GPs, and nearly one third reported using amoxicillin and clavulanate.

### Strengths and limitations of the study

Conducting online surveys with health professionals has several benefits thereby making the web-based approach an attractive alternative to postal or telephone methods as they are easy to be implemented and allow large-scale surveys without postage costs [15]. Moreover, electronic surveys allow tighter control of the order in which respondents respond the different questions, thus preventing respondents from going back and changing their answers. Another advantage of electronic questionnaires is that data is automatically transferred to a database, thereby eliminating the need to enter the data manually and avoiding potential errors of data entry. However, the major obstacle is external validity, and specifically how to obtain a representative sample and adequate response rate [16]. As acknowledged in other studies, respondents to electronic surveys are usually unrepresentative of the whole community of health professionals even within a certain specialty such as primary care. In an online survey of a group of 800 web-using doctors in the UK, female doctors were found to be significantly underrepresented [17]. One cannot assume that registered members of a specific web site will necessarily reflect the whole group of these health professionals. In our study, the questionnaire was available in two scientific societies’ webpages and, in addition, demographic data were not requested in this questionnaire and therefore information about age and gender of the respondents is lacking. However, GPs were allowed to answer only once. The internet-based questionnaire may have introduced selection bias, as only internet users were invited to participate in the study. However, nearly all the GPs now have access to internet, at least in their workplace, since all the computers have access to internet throughout the country. Another difference is the gap between opinion and actual practice.

This was a study of opinions and perceptions which does not necessarily reflect the actual number of consultations due to acute pharyngitis and the real prescription habits. GPs clearly overestimated the number of acute episodes of sore throat as they admitted that this was the reason of encounter in one quarter of the consultations. This figure significantly contrasts with the percentages of episodes of acute pharyngitis reported in epidemiologic surveys. For instance, in a year-round study in two Spanish practices acute pharyngitis accounted for nearly 15% of the consultations due to an infectious disease [1]. In other studies, carried out in other countries, this percentage is even lower, ranging from 2 to 4% of the consultations seen by a family doctor [18, 19]. The present study clearly overestimates the physicians’ perceptions of how many episodes of acute pharyngitis they have retrospectively visited. Conversely, they tend to underestimate the percentages of cases in which an antibiotic is given [20]. As in other studies of opinions the results obtained do not accurately reflect what is in fact prescribed in primary offices, but rather correspond to hypothetical scenarios in which GPs were simply asked to give their opinion about each specific situation. This might explain why we only observed 32% of GPs who would prescribe antibiotics in the second case, describing a patient with two Centor criteria. Notwithstanding, on the basis of the results obtained in audit-based studies, a percentage ranging from 40 to 60% of the cases of pharyngitis and tonsillitis are being treated with antibiotics in Spain [8, 21]. However, in terms of what antibiotic would be used, the results obtained in this questionnaire-based study are similar to those obtained in other studies [8]. Another limitation, inherent to the Spanish healthcare system, is that we only considered when GPs think an antibiotic is warranted; however, since over-the-counter sale of antibiotics is still available, the actual consumption of antibiotics might be much higher [22].

Despite these weaknesses, this manuscript provides some insights about how GPs manage the treatment of cases of acute pharyngitis with the use of two clinical scenarios. One strength of this electronic survey, however, is the fact that this is one of the largest studies on public views of management of sore throat in Europe, despite only 5% of the members of both societies answering the questionnaire.

### Comparison with existing literature

Patients with less than two Centor criteria have less than a 10% probability of having pharyngitis caused by group A β-haemolytic streptococcus while this probability ranges from 10 to 17% in patients with two criteria [3, 23]. Clinical guidelines endorsed by the most prestigious societies, such as the Infectious Diseases Society of America, the National Institute for Health and Clinical Excellence and the European Society of Clinical Microbiology and Infectious Diseases, do not recommend the utilisation of antibiotics for patients with sore throat and less than two Centor criteria [24–26]. Despite the fact that guidelines also recommend the use of antibiotics in bacterial infections other than group A streptococcus, such as gonococcal infection, its incidence is very low in the primary care setting.

In a review of guidelines on sore throat in Europe and North America, Matthys et al. observed that of the ten national guidelines analysed, recommendations differed with regard to the use of a rapid antigen detection test and throat culture and with the indication for antibiotics. The North American, French, and Finnish guidelines considered diagnosis of GAS essential. In 4 of the 6 European guidelines, acute sore throat was considered a self-limiting disease and antibiotics were not recommended [27]. However, the review of the Cochrane Library recommends only the use antibiotics when streptococcal infection is suspected, since it has been demonstrated that antibiotic therapy reduces the number of complications [5]. Recently, Little et al. observed few complications in pharyngitis not treated with antibiotics, only 1.3% [28]. Most of the clinical guidelines, including those issued by the Infectious Society of North America and the Spanish guideline endorsed by GPs, recommend the use of rapid antigen detection tests for patients with two or more Centor criteria. Even patients with all the criteria described by Centor et al. – the four criteria – have a probability of streptococcal infection ranging from 39 to 57%. However, the adherence of Spanish GPs to these recommendations is very poor, since only one out of ten clinicians use the point-of-care tests to better identify the GAS in their consultations.

One of the limitations of GPs not using rapid tests is the uncertainty of the aetiological agent and this can lead to antibiotic overprescribing. The choice of empiric antimicrobial chemotherapy is guided in these cases by the clinical presentation, the severity of the infection, and epidemiologic data, comprising the causative organisms and their susceptibility to antimicrobial agents. However, GPs usually overestimate the incidence of streptococcal infection when the management is only based on clinical grounds. Due to the severity of these infections and the difficulties in determining the streptococcal aetiology, treatment is often empirical, usually consisting of orally administered agents.

According to the results obtained in this study two great areas of improvement arise: first, the use of antibiotics for otherwise suspected viral sore throat is considerable, as nearly one in five clinicians declared treating these patients unnecessarily. Second, there is also room for improvement regarding the choice of antibiotics in suspected or confirmed cases of streptococcal infection, since only 12% of the respondents stated that they would treat these infections with penicillin V. We found that more than nearly one third of the GPs preferred the prescription of a broad-spectrum β-lactam, such as amoxicillin and clavulanate. However, GAS is highly susceptible to penicillin (strains with a minimum inhibitory concentration >0.12 μg/mL have not been found) and should be considered susceptible to all β-lactams [29]. In some regions of the world, streptococcal resistance to antibiotics such as macrolides, clindamycin, and lincosamide has become an increasing concern [30, 31]. In other studies carried out in Spain, the use of antibiotics other than penicillin V, have ranked first in pharyngitis, with amoxicillin being the leading antibiotic used, as in our study [32, 33]. However, in an older study, amoxicillin and clavulanate was the preferred antibiotic by Spanish GPs [34].

Another issue to be highlighted in this study is the use of treatments other than antibiotics for patients with sore throat. Both oral analgesics and non-steroidal anti-inflammatory drugs were recommended by more than half of the respondents. Guidelines do not usually specify what drug is recommended and neither does the dose of these drugs suggested. However, most of the GPs stated to use usual doses of non-steroidal anti-inflammatory drugs and this has been shown to be associated with a higher risk for gastrointestinal disturbances [35]. It is also curious to observe that GPs are more prone to prescribe other drugs, such as decongestants, antitussives, expectorants and herbal remedies, when the infection is highly likely to be caused by a virus, a feature that has not been observed in other publications. These drugs are only available over-the-counter and have not shown to be effective. However, some clinicians might feel forced to give or recommend some products, mainly when an antibiotic is not prescribed.

## Conclusions

In conclusion, this paper highlights an array of misconceptions about the use of rapid antigen detection tests and about the type and indications of drugs, mainly antibiotics, which GPs have in the management of sore throat. These results suggest that guidelines on this very common self-limiting condition are seldom followed by clinicians.

## Abbreviations

GAS: Group A streptococcus
GP: General practitioner
Semergen: *Sociedad Española de Médicos de Atención Primaria* [Spanish Society of Primary Care Physicians]
SemFYC: *Sociedad Española de Medicina de Familia y Comunitaria* [Spanish Society of Family and Community Medicine]
